# Respiratory and non‐respiratory airflow characteristics across ingestive and non‐ingestive swallowing tasks

**DOI:** 10.1113/EP092561

**Published:** 2026-03-18

**Authors:** Elizabeth Cross, Esther Guiu Hernandez, Phoebe Macrae

**Affiliations:** ^1^ Rose Centre for Stroke Recovery and Research University of Canterbury Christchurch New Zealand

**Keywords:** deglutition, mouth breathing, oronasal, respiratory phase patterns, respiratory–swallowing coordination, swallowing

## Abstract

Respiratory–swallowing coordination is critical for safe swallowing. Respiratory phase patterns, indicating the direction of respiration bracketing swallowing, appear minimally altered when oral airflow is measured alongside nasal airflow. Measures from an oronasal facemask also demonstrate broader features of swallowing non‐respiratory flow (SNRF) associated with the respiratory–swallowing pause than previously reported. Additionally, nasal and oronasal flow estimates show variability in within‐individual respiratory phase patterning. In this study, an oronasal facemask and separate nasal‐only mask were used to assess healthy individuals, exploring how instrumentation influences respiratory phase pattern estimates across swallowing tasks. Characteristics of SNRF and variability of respiratory phase patterns were also investigated. Adding oral flow to nasal flow appeared to reduce estimated exhale–swallow–exhale patterning by 13% and increased the estimated frequency of alternative patterns, although this may include some genuine variance between the datasets due to asynchronous data acquisition from each mask. SNRF occurred in 87–97% of swallows, with inward and outward flow occurring at the onset and offset of the respiratory–swallowing pause. Test–retest reliability of respiratory phase patterns within individuals ranged from κ = 0.4 ‐ 0.54, with ≥2 patterns observed across four trials >40% of the time. Findings suggest that adding oral to nasal flow alters estimates of respiratory phase patterns, particularly during ingestive swallowing tasks. SNRF is observed more frequently when measured through a closed system, presenting as inwardly‐ or outwardly‐directed airflow at the beginning or end of the respiratory–swallowing pause. Finally, there appears to be considerable variability in respiratory phase patterning across trials of the same swallowing condition.

## Abstract (highlights)

- **What is the central question of this study?** Are estimates of respiratory phase patterns influenced by the addition of oral airflow data to nasal airflow data during assessment of respiratory–swallowing coordination?
- **What is the main finding and its importance?** Estimates of respiratory phase patterns are altered when oral airflow data are captured alongside nasal airflow data, particularly when assessing ingestive swallowing tasks. These findings demonstrate the importance of accounting for all respiratory airflow (i.e., nasal and oral) in assessment of respiratory–swallowing coordination, particularly in populations prone to mouth breathing.

## 1 INTRODUCTION

Sound respiratory–swallowing coordination (RSC) is essential for the safe ingestion of food and fluids. Respiratory phase patterns are typically characterized by the direction of respiratory airflow before and after the respiratory–swallowing pause, with patterns including exhale–swallow–exhale (EX–EX), inhale–swallow–exhale (IN–EX), exhale–swallow–inhale (EX–IN) and inhale–swallow–inhale (IN–IN). Swallowing during expiration (EX–EX) and at mid‐low quiet breathing lung volumes is considered optimal, offering advantages to safety and biomechanics (Martin‐Harris et al., 2023; McFarland et al., 2016), with EX–EX patterning widely shown to be the most common pattern in healthy individuals (Hopkins‐Rossabi et al., 2019; Martin et al., 1994; Martin‐Harris et al., 2005, 2003; Perlman et al., 2000; Preiksaitis & Mills, 1996; Preiksaitis et al., 1992). Deviations in respiratory‐phase patterning have been demonstrated in multiple populations including head and neck cancer (Brodsky et al., 2010; Hopkins‐Rossabi et al., 2021), Parkinson's disease (Gross et al., 2008; Troche et al., 2011) and chronic obstructive pulmonary disease (Gross et al., 2009). In head and neck cancer, the severity of swallowing impairment (dysphagia) has been shown to correlate with disrupted respiratory phase patterning (Brodsky et al., 2010) and increased incidence of non‐EX–EX swallows (Hopkins‐Rossabi et al., 2021). Occurrence of penetration and aspiration of fluids into the airway during ingestion has been linked to post‐swallow inhalation in those with Parkinson's disease (Troche et al., 2011), and inconsistent respiratory phase patterning or non‐EX–EX patterning (Brodsky et al., 2010) in head and neck cancer. In response to known alterations in respiratory phase patterning, RSC‐based treatments are emerging (Curtis, Dakin et al., 2020, 2020; Martin‐Harris et al., 2015) as a management approach in dysphagic individuals. However, such treatments may be somewhat limited by the lack of understanding of normal respiratory phase pattern variability in healthy individuals.

While several foundational RSC studies have used a nasal cannula to assess respiration through the nose (Martin‐Harris et al., 2005, 2003), the use of nasal respiratory measures in isolation may not be sensitive to the respiratory behaviour of mouth breathers, as incidentally demonstrated by Gross et al. (2009). Mouth breathers are known to breathe simultaneously through the nose and mouth, in contrast to most healthy individuals who breathe solely through the nose (Nascimento et al., 2019; Niinimaa et al., 1981). A recent study measuring nasal respiratory flow and combined oronasal flow with a partitioned oronasal facemask found that adding oral to nasal flow (thus, reflecting the behaviour of a mouth breather) minimally altered estimates of respiratory phase patterns, particularly for 10 mL water boluses (Cross et al., 2024). However, it remains unknown whether the difference between nasal and oronasal estimates reflected a true difference in estimated respiratory patterns due to occurrence of habitual mouth breathing, or if the cumbersome oronasal facemask caused measurement error or caused participants to behave atypically. Oronasal flow estimates from the partitioned oronasal facemask need to be compared to isolated nasal flow estimates from a distinct measurement system to determine the relative contribution of the instrumentation used, including whether data were truly altered by addition of oral flow and whether participants’ breathing and swallowing were altered by the use of the oronasal facemask.

Additionally, respiratory data collected using the oronasal facemask revealed that non‐respiratory flow associated with swallowing may present in a manner broader than previously recognized (Cross et al., 2024). Swallowing non‐inspiratory flow (SNIF), a brief inward non‐respiratory flow event at the offset of the respiratory–swallowing pause (Brodsky et al., 2012; Martin‐Harris et al., 2003; Paydarfar et al., 1995), has previously been observed in 67–74% of participants across two swallowing trials (Brodsky et al., 2012). Videofluoroscopic investigations support the notion that SNIF results from re‐opening of the pharynx after swallowing (Brodsky et al., 2012; Paydarfar et al., 1995). Flow appears to occur concurrently with downward movement of the velum (Brodsky et al., 2012), separation of the base of tongue from the posterior pharyngeal wall (Brodsky et al., 2012) and offset of pharyngeal contraction (Paydarfar et al., 1995). Although early investigations suggested that laryngeal valving continued past the SNIF (Paydarfar et al., 1995), subsequent evidence has suggested that flow coincides with the onset of airway re‐establishment (Brodsky et al., 2012; Martin‐Harris et al., 2003). Regardless, the fact that SNIF appears to occur right at the onset of airway opening, before it is fully patent, supports the notion that flow is non‐respiratory (Brodsky et al., 2012; Paydarfar et al., 1995). Expanding on previous reporting of SNIF, recent investigations have indicated that non‐respiratory flow events may not be limited to inward flow at the offset of the respiratory–swallowing pause (Cross et al., 2024). New evidence has demonstrated non‐respiratory flow events occurring at the onset of the pause as well as the offset, which may consist of outwardly‐directed airflow (‘non‐expiratory’) as well as inward (Cross et al., 2024). The revised term *swallowing non‐respiratory flow* (SNRF) was coined to reflect these new findings (Cross et al., 2024). The differences in observed presentation of this phenomenon were likely detected due to use of a system that fully sealed the mouth and nose, therefore increasing sensitivity to low‐amplitude flow events compared to nasal cannula used to date (Brodsky et al., 2012; Martin‐Harris et al., 2003). As a result, the characteristics of SNRF require further investigation with more rigorous physiological measures. Although increased measurement sensitivity brings potential benefit, for example, by revealing previously unrecognized features of SNRF, there is also a risk of increasing ambiguity and thus, reducing scientific accuracy. It is essential to determine whether our previous data are likely to provide a more accurate representation of SNRF, or whether the instrumentation simply caused measurement error. This will not only advance understanding of the characteristics of SNRF, but will also inform methods for future investigations.

The finding of low within‐subject, within‐session test–retest reliability of respiratory phase patterns in a healthy population (Cross et al., 2024) suggests considerable variability exists in respiratory behaviour surrounding swallowing. Whilst previous research reports no significant difference in the prevalence of EX–EX swallows across two trials of the same bolus type (Brodsky et al., 2010; Martin‐Harris et al., 2005, 2003), this is based on group estimates and does not necessarily reflect stability at the individual level. Although no other studies have directly investigated test–retest reliability in a healthy cohort, there are multiple reports indicating that a degree of variability may exist in respiratory phase patterns (Hardemark Cedborg et al., 2009; Wheeler Hegland et al., 2011, 2009). In spontaneous dry swallows, a third (*n* = 2) of participants used two respiratory phase patterns, EX–EX and EX–IN (Hardemark Cedborg et al., 2009). Use of multiple respiratory phase patterns has been observed within participants across numerous bolus trials (Wheeler Hegland et al., 2009), indicating a degree of variability across trials, though this may have been impacted by differing bolus consistencies. During continuous drinking trials, 70% (*n* = 14) of participants reportedly used all four respiratory phase patterns, while the remaining 30% (*n* = 6) used at least two patterns across swallows (Wheeler Hegland et al., 2011).

This study had three aims. The first aim was to determine if differences between nasal and oronasal respiratory data (Cross et al., 2024) are likely to reflect differences in respiratory phase patterning in individuals prone to habitual mouth breathing, or whether the previously‐used oronasal facemask (a) incorrectly measured airflow or (b) caused participants to behave differently during experimental swallowing tasks. The impact of oral airflow on respiratory phase patterning was examined by comparing oronasal flow measures (collected using an oronasal facemask) to nasal flow measures (collected using a separate nasal‐only mask) in order to determine the differential effects of capturing all airflow. The second aim was to determine whether inward and outward non‐respiratory flow events observed at the beginning and end of the respiratory–swallowing pause (Cross et al., 2024) are likely to be a true characteristic of SNRF. Abdominal kinematic data were used to supplement nasal and oronasal airflow measures, thus allowing for improved distinction between respiratory and non‐respiratory airflow. The final aim was to explore the consistency (test–retest reliability) of respiratory phase patterns across an increased number of swallowing trials, to provide more thorough data on intra‐individual variability compared to previous work (Cross et al., 2024).

## 2 METHODS

### 2.1 Participants

Ethical approval for this research was granted by the institutional ethical review board (reference: HREC 2022/73/LR‐PS). The study was completed in accordance with the *Declaration of Helsinki*. Individuals were included if they were 18 years or older and healthy; they were excluded if they had a history of dysphagia or respiratory conditions, with the exception of well‐controlled asthma. The sample size was calculated based on the number of swallowing trials required for intra‐ and inter‐rater reliability analyses. This was determined to be 37 swallowing trials per condition (8 conditions at most, for a total of 296 trials; K1 = 0.3, K2 = 0.6, power 80%, 𝛼 0.05); the total was then doubled (74 trials per condition, with 592 trials total) to account for the expected disproportionality of respiratory phase patterns within the dataset (Bujang & Baharum, 2017) caused by EX–EX predominance (Hopkins‐Rossabi et al., 2019). Additionally, four trials of each swallowing condition were sought to allow some variation in respiratory phase patterns to be observed. At four trials per swallowing condition per participant, across a total of 25 participants, an expected total of 100 swallows per condition met sample size requirements.

### 2.2 Instrumentation

Combined oronasal respiratory flow was detected using an oronasal facemask, with partitioning between the oral and nasal ports (Dual Port Silicone Rubber Face Mask; Hans Rudolph Inc., Shawnee, KS, USA). The facemask was modified to allow insertion of a straw, as previously described (Cross et al., 2024). A spirometry flow head (MLT300L, ADInstruments, Dunedin, New Zealand) was attached to each port of the facemask via a custom‐made 3D‐printed tube and a filter (SureGard, Bird Healthcare, Bayswater, VIC, Australia). The flow heads were connected to two spirometers (FE141, ADInstruments) via the supplied tubing. Nasal respiratory flow was assessed using a nasal continuous positive airway pressure (CPAP) mask (AirFit N20 Nasal Mask, ResMed, San Diego, CA, USA). The mask was attached to a single spirometry flow head (MLT300L, ADInstruments) using a filter (SureGard, Bird Healthcare) and silicone tube, with the spirometry flow head connected to the spirometer using supplied tubing. Prior to each data collection session, the spirometers were calibrated separately for the oronasal and nasal masks using a 3 L calibration syringe (Medikro 3000, M9474, Medikro, Kuopio, Finland). Respiratory bands were used to supplement flow measures with estimates of chest (TN1132/ST, ADInstruments) and abdominal (Pneumotrace II™, model unknown, UFI, Morro Bay, CA, USA) movement, associated with respiration. Both bands were connected to PowerLab® via the Quad Bridge Amp (FE224, ADInstruments). Surface electromyography (sEMG; Trigno Wireless EMG system; Delsys, Natick, MA, USA) was used to detect submental muscle activity associated with swallowing. Signals from the spirometers, respiratory bands and sEMG were all recorded using PowerLab® hardware (PowerLab® 8/35‐2264; ADInstruments). Spirometry was sampled at a rate of 100/s, with a range of 500 mV. Signals were analysed using LabChart (version 8, ADInstruments) and the LabChart® spirometry extension (Spirometry Analysis Software, version 2.5.3, ADInstruments). A pre‐set low‐pass analog filter of 100 Hz was used as part of the spirometry extension. sEMG signals were band‐pass filtered between 10 and 500 Hz (De Luca et al., 2010; Stepp, 2012).

### 2.3 Procedures

Participants attended one assessment session at a swallowing research laboratory. They were seated for the entirety of the session. sEMG electrodes were adhered to the skin overlying the submental muscles, with a ground electrode placed over the left sternum. The respiratory bands were worn around the chest at mid‐sternal level (McFarland et al., 2016) and around the abdomen at the level of the umbilicus (Wheeler Hegland et al., 2011, 2009). Participants underwent the assessment protocol twice, once while wearing the oronasal facemask and once while wearing the nasal mask. The order in which the masks were worn was counterbalanced across participants. The masks and spirometer units were suspended from a rod above the participant's head. The height of the rod was adjusted to ensure consistency of posture throughout the procedure and to take the weight of the unit off the participant. Each mask was secured to the head using adjustable headpieces supplied with the masks.

Participants were instructed to sit relaxed and read from a supplied magazine or their phone, and to refrain from talking for 10 minutes. This was done to distract them from their breathing and swallowing, with the aim of increasing naturalness, as spontaneous swallows were assessed during this time, unbeknown to participants. Following the rest period, participants completed four cued dry (saliva) swallows. These were cued by the researcher using the phrase ‘swallow whenever you're ready’. At least one full respiratory cycle was completed (as observed on LabChart®) between completion of one swallow and cuing of the next. Cuing was not supplied at a particular point in the respiratory cycle to avoid influencing the participant's behaviour. After completion of the dry swallowing trials, a range of ingestive swallowing trials were completed in an order counterbalanced across participants. A straw was used for water delivery during discrete and continuous drinking trials. When using the oronasal facemask, the straw was inserted into the mouth through a hole in the facemask and held in the mouth by the participant (the hole was plugged during other tasks to prevent airflow leakage). During nasal mask trials, the straw was held in the mouth by the participant. Discrete 10 mL water boluses were syringed into the straw by the researcher. Four trials were completed with a bolus hold prior to swallowing, using the instructions ‘hold the water in your mouth, count to one [in your head], and then swallow’. Participants were instructed to raise their hand when they swallowed. Hand raises were marked online in LabChart® by the researcher. Four discrete 10 mL water swallows were completed without a bolus hold (immediate swallowing), using the instructions ‘swallow as soon as you feel the water in your mouth’. Due to the predictability of the immediate swallowing condition, a hand raise was not required; instead, swallows (as observed by the researcher based on laryngeal elevation and/or audible swallowing sounds) were marked online in LabChart®. For continuous drinking trials, participants were provided with a cup filled with 150 mL of water and instructed to ‘drink this in one go, at a speed that is comfortable for you’. Swallows were marked online in LabChart®, as observed by the researcher based on laryngeal elevation and, when present, audible swallowing sounds. For cracker trials, participants were given four halves of an Arnott's Salada™ cracker segment to eat one at a time. During oronasal mask trials, the researcher unfastened the mask at the side, before placing the cracker into the participant's mouth. They were instructed to hold the cracker in the mouth without chewing until the facemask was re‐fastened. Participants were able to self‐feed during the nasal mask trials. Instructions were ‘chew the cracker, then swallow when you are ready, and raise your hand when you swallow’. Hand raises were again marked in LabChart® for offline analysis. The final two conditions, involving drinking from a cup, were only assessed with the nasal mask, due to the inability to drink from a cup while wearing the oronasal facemask. Participants were provided with a small Flexi Cup filled with water. Four cup drinking trials were completed with a bolus hold. Participants were instructed to ‘take a normal sized sip from the cup, hold it in your mouth, count to one [in your head] and then swallow and raise your hand’. Hand raises were marked in LabChart®. Four trials were completed without a bolus hold (immediate swallowing). For immediate swallowing trials, participants were instructed to ‘take a normal sized sip from the cup and swallow the water straight away’. Swallows (as observed based on laryngeal elevation and/or audible swallowing sounds) were marked in LabChart®.

### 2.4 Data analysis

For analysis of oronasal data, the spirometry signals from the separated nasal and oral channels of the facemask were summed in LabChart®, providing an estimate of combined flow from the mouth and nose. Isolated nasal data were analysed from data collected using the nasal‐only mask. A positive polarity waveform indicated inhalation, while a negative polarity waveform indicated exhalation. Respiratory–swallowing pause periods were indicated via a plateau at zero in the respiratory waveform.

All swallows were identified based on a simultaneous pause in the respiratory waveform and a burst of sEMG activity indicative of submental muscle activation. For spontaneous swallows, the first four swallows to occur in the rest period were analysed. Swallows from all other conditions (dry, 10 mL bolus hold, 10 mL immediate, continuous, cracker, cup bolus hold, cup immediate) were determined based on a simultaneous respiratory–swallowing pause, a burst of sEMG activity, as well as a comment in LabChart® indicating either (a) a hand raise by the participant or (b) swallowing observed by the researcher. The first four swallows to occur during the continuous drinking trial were analysed, regardless of whether they occurred in one or more ingestive cycles (Dozier et al., 2006); when multiple sequential swallows occurred within a single ingestive cycle, each swallow within that cycle was assigned the same respiratory phase pattern depending on the direction of respiration before and after the set of swallows. Individual swallows were numerically labelled in preparation for data analysis, ensuring that the raters analysed the same point in the waveform. Additionally, this allowed the raters to be blinded to the swallowing condition, although the continuous swallowing condition may have been identifiable by the raters due to the proximity of swallows.

Respiratory phase patterns were coded according to the direction of respiratory airflow (from either oronasal or nasal data) before and after swallowing, that is, EX–EX, IN–EX, EX–IN or IN–IN. When a SNRF event was detected (either at the very beginning or very end of the respiratory–swallowing pause), SNRF was coded as ‘present’; additionally, direction (‘inward’ for non‐inspiratory flow, or ‘outward’ for non‐expiratory flow) and timing (‘onset‐pause’ at the beginning of the respiratory–swallowing pause, ‘offset‐pause’ at the end, or ‘both’) were recorded. If no SNRF event was detected, SNRF was coded as ‘absent’. Respiratory phase patterns and SNRF were primarily identified using the respiratory flow channels, but measures of abdominal kinematics were used to aid interpretation when the flow channel was unclear (typically due to SNRF). If abdominal movement reflected the same respiratory patterning as the flow channel, the flow event was considered to be caused by true respiration. When abdominal movement did not match the flow channel, the flow event was considered to be SNRF. Chest movement estimates had to be excluded from data analysis due to technical difficulties discovered during data collection. This may have reduced the sensitivity of the kinematic measures, as displacement of the abdomen is less than that of the chest during respiration; however, the placement of the abdominal band over the lower rib cage means that it is still likely to detect a degree of rib cage movement (Banzett et al., 1995). Additionally, as swallowing occurs during tidal breathing (McFarland et al., 2016), an increased degree of rib cage displacement was not expected. As such, it was deemed appropriate to use abdominal kinematic data during analysis. A specific airflow or kinematic threshold for distinguishing between respiratory flow and SNRF was not set, as such a threshold has not been determined for the instrumentation used in this study, making any threshold value arbitrary. The LabChart® display was set to the same dimensions for analysis of each file (20:1 horizontal scaling) to ensure consistency across participants. Channels were auto scaled before each individual trial was coded, so that the waveform was displayed relative to surrounding airflow.

A total of 966 swallowing trials were used to determine intra‐ and inter‐rater reliability. Overall, 69 trials from each mask and each swallowing condition were re‐analysed by the first author (intra‐rater) and by a trained research assistant (inter‐rater).

### 2.5 Statistical analysis

#### 2.5.1 Intra‐ and inter‐rater reliability

Intra‐ and inter‐rater reliability were calculated using Cohen's κ (95% CI), with the expected predominance of EX–EX trials (see Hopkins‐Rossabi et al., 2019; Martin‐Harris et al., 2005) likely causing low κ estimates due to the kappa paradox (Byrt et al., 1993; Feinstein & Cicchetti, 1990) and, therefore, misrepresenting reliability. Therefore, a prevalence and bias adjusted κ (PABAK; 95% CI) and percentage of agreement (POA) were also calculated. Values were calculated for each swallowing condition individually for each mask, as well as all conditions combined for each mask.

#### 2.5.2 Frequency of respiratory phase patterns across masks

To compare respiratory phase patterns across the nasal and oronasal methods, the frequency of each pattern (raw number of occurrences, and percentage out of all swallows) was determined descriptively using the full dataset (1354 swallows, as rated by the first author). The percentage of each respiratory phase pattern for each condition separately and all conditions combined were compared between the nasal and oronasal masks. Because data were collected at different time points and with different masks, results for each trial could not be directly compared, meaning comparisons were completed using the full dataset, with some variance expected. In order to assess the effect of drinking through a straw, the frequency of each respiratory phase pattern was compared between cup and straw trials, with and without a bolus hold, from the nasal‐only data.

#### 2.5.3 SNRF

The raw number of SNRF occurrences and the percentage of swallows in which SNRF occurred were calculated. Additionally, descriptive analyses were used to determine the raw number and percentage of inward (‘non‐inspiratory’) and outward (‘non‐expiratory’) SNRF events at the beginning (onset) and end (offset) of the respiratory–swallowing pause. Post hoc analyses were completed to assess the intra‐ and inter‐rater reliability of SNRF data, including presence/absence, position (onset‐ or offset‐pause), and direction (inward or outward). Cohen's κ (95% CI), PABAK (95% CI) and POA were calculated for all SNRF events from the nasal and oronasal data.

#### 2.5.4 Test–retest reliability

To calculate the test–retest reliability (within‐session, within‐subject) across a set of four trials of the same swallowing condition, POA was averaged across paired trials (aPOA). Additionally, PABAK was averaged (aPABAK) across paired trials to create a prevalence and bias‐adjusted Light's κ. The 95% CI was calculated using bootstrapping. Test–retest reliability was determined using aPOA and aPABAK for the full respiratory phase patterns (EX–EX, IN–EX, EX–IN, IN–IN); the same were also used for post hoc analysis of post‐swallow respiratory flow direction (i.e., inhalation or exhalation) due to observations of greater stability of airflow direction post‐swallow. Descriptive analyses were also completed post hoc to determine how many, and which, respiratory phase patterns were used across a set of four trials of the same swallowing condition.

## 3 RESULTS

A total of 26 participants (14 female) were recruited for the study and provided informed, written consent. In the oronasal condition, a total of 624 trials were collected across the six swallowing conditions. The first author excluded 51 swallows prior to data analysis due to (a) excessive artifact that affected identification of respiratory flow or (b) excessive artifact in the sEMG signal that caused ambiguity as to whether a swallow had occurred. This left 573 oronasal trials for further analysis. In the nasal condition, 832 trials were collected, with 51 excluded for the same reasons, leaving a total of 781 nasal trials. Across the oronasal and nasal conditions combined, there was a total of 1354 swallowing trials.

### 3.1 Intra‐ and inter‐rater reliability

Intra‐ and inter‐rater reliability data are presented for each individual swallowing condition, and all conditions combined, in Table 1. Either the Cohen's κ or PABAK value was selected for interpretation, with PABAK used when a prevalence problem was detected in the dataset (as indicated with bold text in Table 1). For the combined conditions, intra‐rater reliability ranged from 0.85 (0.79, 0.9; oronasal) to 0.97 (0.94, 0.99; nasal). Inter‐rater reliability for all conditions combined was 0.85 (0.8, 0.91) for the oronasal data but only 0.57 (0.49, 0.64) for nasal data.

**TABLE 1:** Intra‐ and inter‐rater reliability.

		All	Dry	10ml hold	10ml imm	Spon	Cracker	Cont	Cup hold	Cup imm
Nasal intra	POA	78%	8%	8%	71%	88%	81%	76%	96%	95%
	Cohen's κ (95% CI)	0.5 (0.42, 0.58)	0.49 (0.24, 0.74)	0.46 (0.2, 0.72)	0.38 (0.15, 0.62)	0.68 (0.47, 0.89)	0.55 (0.33, 0.77)	**0.62 (0.46, 0.78)**	0.84 (0.62, 1.06)	0.68 (0.26,1.04)
	p‐value	1.475	<0.001	<0.001	0.001	2.437	8.400	3.928	3.375	0.001
	PABAK (95% CI)	**0.57 (0.49, 0.64)**	**0.61 (0.37, 0.78)**	**0.61 (0.37, 0.78)**	**0.42 (0.17, 0.63)**	**0.75 (0.54, 0.89)**	**0.62 (0.4, 0.79)**	0.53 (0.29, 0.72)	**0.93 (0.75, 0.99)**	**0.9 (0.73, 0.98)**
Oronasal intra	POA	93%	92%	93%	97%	97%	94%	83%		
	Cohen's κ (95% CI)	**0.85 (0.8, 0.91)**	0.76 (0.56, 0.96)	**0.86 (0.74, 0.98)**	**0.95 (0.87, 1.02)**	0.9 (0.76, 1.04)	0.72 (0.46, 0.99)	**0.77 (0.65, 0.9)**		
	p‐value	4.477	1.219	3.822	1.006	1.169	6.206	2.429		
	PABAK (95% CI)	0.85 (0.79, 0.9)	**0.85 (0.66, 0.95)**	0.85 (0.67, 0.95)	0.94 (0.78, 0.99)	**0.94 (0.79, 0.99)**	**0.87 (0.69, 0.96)**	0.67 (0.44, 0.83)		
Nasal inter	POA	98%	100%	100%	98%	100%	100%	97%	96%	95%
	Cohen's κ (95% CI)	0.95 (0.91, 0.98)	1 (1, 1)	1 (1, 1)	0.95 (0.84, 1.05)	1 (1, 1)	1 (1, 1)	**0.94 (0.87, 1.02)**	0.84 (0.62, 1.06)	0.65 (0.26, 1.04)
	p‐value	<0.001	<0.001	<0.001	1.766	<0.001	<0.001	2.001	3.375	0.001
	PABAK (95% CI)	**0.97 (0.94,0.99)**	**1 (0.89, 1)**	**1 (0.89, 1)**	**0.97 (0.84, 1)**	**1 (0.89, 1)**	**1 (0.90, 1)**	0.94 (0.80, 0.99)	**0.93 (0.75, 0.99)**	**0.9 (0.73, 0.98)**
Oronasal inter	POA	93%	95%	99%	94%	98%	94%	79%		
	Cohen's κ (95% CI)	**0.85 (0.79, 0.9)**	0.8 (0.58, 1.02)	**0.97 (0.91, 1.03)**	**0.89 (0.79, 0.99)**	0.95 (0.84, 1.05)	0.48 (‐0.01, 0.97)	**0.69 (0.55, 0.84)**		
	p‐value	1.377	8.433	1.867	1.799	2.173	0.057	9.671		
	PABAK (95% CI)	0.86 (0.8, 0.91)	**0.91 (0.75, 0.98)**	0.97 (0.84, 1)	0.87 (0.69, 0.96)	**0.97 (0.83, 1)**	**0.87 (0.69, 0.96)**	0.58 (0.34, 0.76)		

*Note*: Cohen's κ = kappa estimate and 95% CI [κ (95% CI)] for rater agreement. Cohen's κ *p*‐value (*p *= 0.05). PABAK = prevalence and bias adjusted kappa estimate and 95% CI [PABAK (95% CI)] for rater agreement. For each condition, either Cohen's κ or PABAK is shown in Bold, with the value in bold indicating that on which the final estimate was based. POA = percentage of agreement.

### 3.2 Frequency of respiratory phase patterns across oronasal and nasal masks

When all swallowing conditions were combined, EX–EX was the most common pattern observed via both the nasal and oronasal masks (82% and 69%, respectively), followed by IN–EX and EX–IN. IN–IN was consistently the least common pattern for both masks across combined swallowing conditions, accounting for less than 3% of swallows overall. The frequencies of each respiratory phase pattern for individual swallowing conditions are displayed in Table 2. The EX–EX pattern was slightly more common in sips from a cup compared the straw, and the frequency of IN–EX swallows increased when the straw was used, although the difference was minimal. The full respiratory phase pattern dataset is supplied as Supporting Information.

**TABLE 2:** Frequency of each respiratory phase pattern.

	EX–EX, *n* (%)	IN–EX, *n* (%)	EX–IN, *n* (%)	IN–IN, *n* (%)	Total
Nasal					
All	637 (82)	83 (11)	54 (7)	7 (1)	781
Spontaneous	78 (83)	5 (5)	11 (12)	0 (0)	94
Dry	84 (84)	15 (15)	1 (1)	0 (0)	100
10 mL hold	82 (82)	16 (16)	2 (2)	0 (0)	100
10 mL immediate	82 (82)	13 (13)	5 (5)	0 (0)	100
Continuous	63 (66)	10 (1 0)	23 (24)	0 (0)	96
Cracker	87 (84)	10 (10)	7 (7)	0 (0)	104
Cup hold	73 (86)	8 (9)	4 (5)	0 (0)	85
Cup immediate	88 (86)	6 (6)	1 (1)	7 (7)	102
Oronasal					
All	393 (69)	104 (18)	61 (11)	15 (3)	573
Spontaneous	70 (81)	9 (10)	7 (8)	1 (1)	87
Dry	84 (85)	11 (11)	4 (4)	0 (0)	99
10 mL hold	61 (61)	32 (32)	7 (7)	0 (0)	100
10 mL immediate	52 (56)	34 (37)	6 (6)	1 (1)	93
Continuous	39 (39)	13(13)	34 (34)	13 (13)	99
Cracker	87 (92)	5 (5)	3 (3)	0 (0)	95

*Note*: the frequencies of each respiratory phase pattern across all swallowing trials combined, and for each swallowing condition, for the oronasal and nasal masks. EX‐EX = exhale‐swallow‐exhale; IN‐EX = inhale‐swallow‐exhale; EX‐IN = exhale‐swallow‐inhale; IN‐IN = inhale‐swallow‐inhale; *n* = number of raw swallows; % = percentage of swallows in a given condition.

### 3.3 SNRF

Intra‐ and inter‐rater reliability of SNRF data are displayed in Table 3 and the full SNRF dataset is supplied as Supporting Information. Intra‐rater reliability suggested good consistency within a single rater when determining if a SNRF event had occurred, with a PABAK of 0.95 (0.91, 0.97) for the nasal data and 0.85 (0.78, 0.89) for the oronasal data. There was poorer inter‐rater consistency, with PABAK values of 0.84 (0.78, 0.88) and 0.78 (0.71, 0.84) for nasal and oronasal data, respectively. As a general rule, reliability was lower when determining the position and airflow direction of SNRF events (Table 3).

**TABLE 3:** Intra‐ and inter‐rater reliability of SNRF measures.

	POA	Cohen's κ	*p‐value*	PABAK
Nasal intra‐				
Presence	97.5	0.43 (0.17, 0.68)	<0.001	**0.95 (0.91, 0.97)**
Position	89	**0.78 (0.73, 0.84)**	<0.001	0.78 (0.72, 0,83)
Direction	91.8	0.63 (0.55, 0.70)	<0.001	**0.84 (0.79, 0.87)**
Oronasal intra‐				
Presence	92.3	0.59 (0.45, 0.72)	<0.001	**0.85 (0.78, 0.89)**
Position	78.4	**0.56 (0.47, 0.64)**	<0.001	0.57 (0.47, 0.65)
Direction	82.7	0.40 (0.31, 0.48)	<0.001	**0.65 (0.58, 0.72)**
Nasal inter‐				
Presence	91.9	0.31 (0.16, 0.46)	<0.001	**0.84 (0.78, 0.88)**
Position	67.5	**0.47 (0.28, 0.45)**	<0.001	0.35 (0.26, 0.43)
Direction	63.0	**0.23 (0.19, 0.27)**	<0.001	0.26 (0.19, 0.33)
Oronasal inter‐				
Presence	89.2	0.59 (0.48, 0.70)	<0.001	**0.78 (0.71, 0.84)**
Position	76.5	0.42 (0.31, 0.52)	<0.001	**0.53 (0.43, 0.62)**
Direction	81.1	0.39 (0.31, 0.46)	<0.001	**0.62 (0.54, 0.70)**

*Note*: Cohen's κ = kappa estimate and 95% CI [κ (95% CI)] for rater agreement. *P*‐value for Cohen's κ (*p *= 0.05). PABAK = prevalence and bias adjusted κ estimate and 95% CI [PABAK (95% CI)] for rater agreement. For each condition, either Cohen's κ or PABAK is in bold, with the value in bold indicating that on which the estimate was based. POA = percentage of agreement.

Using the nasal mask, SNRF was detected on 97% (755) of swallows. Of those swallows, SNRF was detected at onset‐pause only in 1% (8), offset‐pause only in 49% (368), and both onset‐ and offset‐pause in 50% (379). Using the oronasal facemask, SNRF was observed on 87% (499) of swallows. Of those swallows, SNRF was detected at onset‐pause only in 3% (17), offset‐pause only in 71% (355), and both onset‐ and offset‐pause in 25% (127).

**FIGURE 1:**
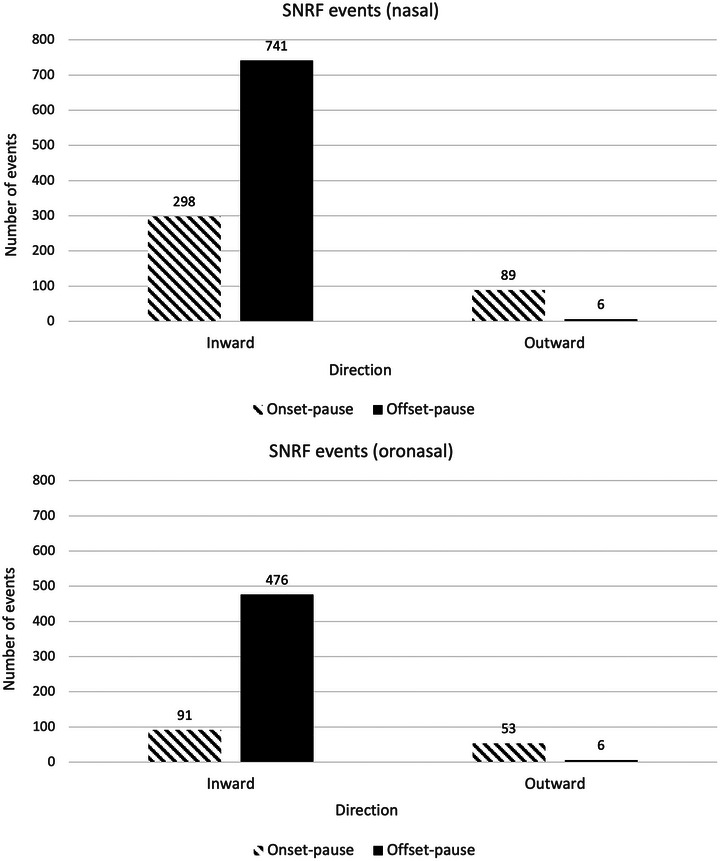
Total SNRF events across nasal and oronasal masks, stratified by direction of airflow (inward or outward) and position in relation to the respiratory–swallowing pause (onset‐pause or offset‐pause).

Of each isolated SNRF event (Figure 1) at onset‐pause, 77% and 63% were inward (298/387 and 91/144; nasal and oronasal), while 23% and 37% were outward (89/387 and 53/144; nasal and oronasal). Of those occurring at offset‐pause, 99% were inward (741/747 and 476/482; nasal and oronasal), and 1% were outward (6/747 and 6/482; for nasal and oronasal).

### 3.4 Test–retest reliability

To determine test–retest reliability, the value of either Cohen's κ or PABAK was selected, depending on whether a prevalence problem was detected in the dataset. When all swallowing conditions were combined, within‐participant test–retest reliability ranged from 0.4 (0.29, 0.5) to 0.54 (0.46, 0.61) for the oronasal and nasal masks, respectively, with an aPOA of 70–77%. Despite our use of a PABAK, the test–retest reliability is likely still underestimated due to the high frequency of EX–EX trials in the dataset; true test–retest reliability may be higher given our aPOA values. aPOA and aPABAK test–retest reliability values for each condition are displayed in Table 4.

**TABLE 4:** Test–retest reliability.

	All	Dry	10 mL hold	10 mL imm	Cont	Cracker	Spon	Cup hold	Cup imm
Nasal full									
aPOA	77%	72%	84%	70%	66%	80%	77%	77%	89%
Lights's κ (95% CI)	0.27 (0.18, 0.39)	−0.03 (−0.12, 0.07)	0.46 (0.16, 0.77)	0.06 (−0.06, 0.26)	0.41 (0.2, 0.68)	0.28 (0.14, 0.43)	0.16 (−0.01, 0.39)	0.11 (−0.01, 0.24)	0.26 (−0.05, 0.43)
*P*	0.769	0.994	0.892	0.983	0.278	0.935	0.975	0.985	0.993
aPABAK (95% CI)	**0.54 (0.46, 0.61)**	**0.44 (0.21, 0.65)**	**0.68 (0.45, 0.86)**	**0.4 (0.18, 0.65)**	**0.32 (−0.01, 0.59)**	**0.6 (0.35, 0.8)**	**0.54 (0.27, 0.74)**	**0.54 (0.22, 0.8)**	**0.78 (0.48, 0.87)**
Nasal post‐pause									
aPOA	91%	98%	97%	91%	75%	93%	88%	94%	98%
Light's κ (95% CI)	0.4 (0.25, 0.57)	^*^	^*^	0.06 (−0.02, 0.11)	0.4 (0.16, 0.67)	0.38 (0.18, 0.5)	0.27 (0.03, 0.47)	^*^	^*^
*P*	0.971	^*^	^*^	1	0.706	0.993	0.989	^*^	^*^
aPABAK (95% CI)	**0.83 (0.76, 0.88)**	**0.96 (0.8, 1)**	**0.94 (0.65, 1)**	**0.82 (0.54, 0.92)**	**0.49 (0.23, 0.9)**	**0.86 (0.62, 0.96)**	**0.76 (0.5, 0.91)**	**0.88 (0.6, 1)**	**0.96 (0.74, 1)**
Oronasal full									
aPOA	70%	76%	59%	64%	61%	87%	72%		
Light's κ (95% CI)	0.37 (0.27, 0.48)	0.11 (−0.03, 0.3)	0.17 (−0.05, 0.48)	0.28 (0.11, 0.57)	**0.45 (0.28, 0.65)**	0.15 (0.08, 0.2)	0.2 (−0.05, 0.42)		
*P*	0.257	0.980	0.799	0.687	0.001	0.996	0.930		
aPABAK (95% CI)	**0.4 (0.29, 0.5)**	**0.51 (0.29, 0.72)**	**0.18 (−0.06, 0.42)**	**0.28 (0, 0.56)**	0.22 (−0.03, 0.47)	**0.74 (0.46, 0.91)**	**0.43 (0.16, 0.7)**		
Oronasal post‐pause									
aPOA	87%	92%	95%	88%	64%	94%	88%		
Light's κ (95% CI)	0.43 (0.29, 0.62)	^*^	^*^	0.29 (0.03, 0.47)	**0.29 (0.06, 0.53)**	0.15 (0.13, 0.15)	0.28 (0.16, 0.39)		
*P*	0.877	^*^	^*^	0.988	0.644	1	0.992		
aPABAK (95% CI)	**0.74 (0.64, 0.81)**	**0.84 (0.58, 0.92)**	**0.91 (0.73, 1)**	**0.76 (0.44, 0.89)**	0.29 (0.06, 0.57)	**0.9 (0.62, 1)**	**0.76 (0.38, 0.88)**		

*Note*: Light's κ = kappa estimate and 95% CI [κ (95% CI), Light's κ *p*‐value (*p *= 0.05), and aPABAK (averaged prevalence and bias‐adjusted κ) with 95% CIs [aPABAK (95% CI)] for test–retest reliability. For each condition, either Light's κ or aPABAK is shown in bold, with the value in bold indicating that on which the estimate was based. ^*^Light's κ and *p*‐value could not be calculated due to lack of spread within the data. aPOA = averaged percentage of agreement.

Test–retest reliability increased when post‐swallow respiratory direction (i.e., inhalation or exhalation) was considered in isolation (i.e., without accounting for pre‐swallow respiratory direction). Overall, post‐swallow test–retest reliability (for all swallowing conditions combined) ranged from 0.74 (0.64, 0.81) with an aPOA of 87% for the oronasal mask to 0.83 (0.76, 0.88) with an aPOA of 91% for the nasal mask.

**FIGURE 2:**
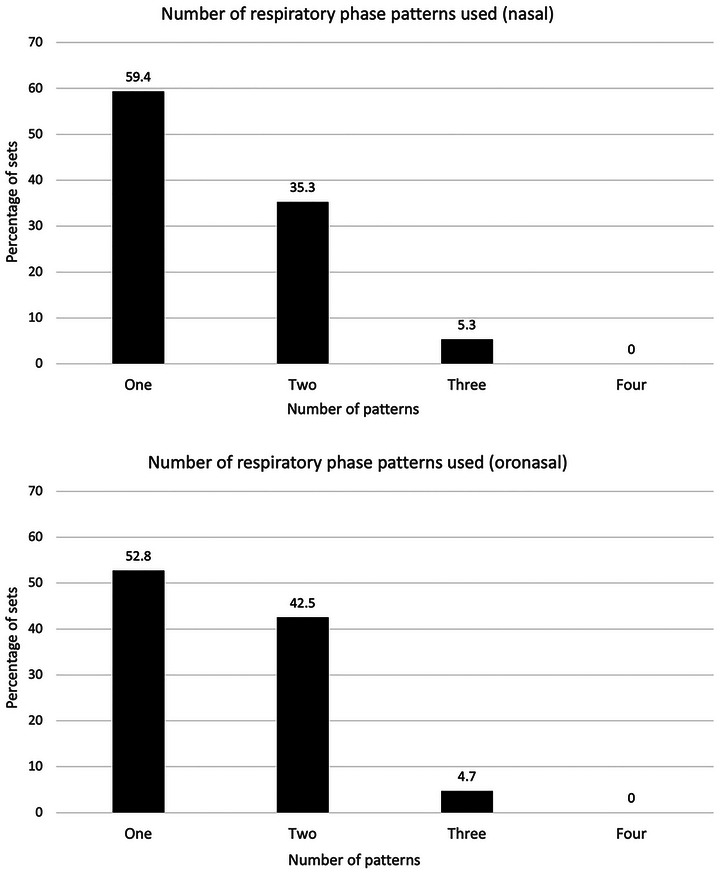
The number of different respiratory phase patterns used by a given individual across four trials of the same swallowing condition, according to the nasal and oronasal data. Number of patterns = the number of respiratory phase patterns observed across any given set of four trials; percentage of sets = percentage (%) of all sets of four trials.

Data revealed that participants used a single respiratory phase pattern across a set of four trials of a given condition 59.4% (nasal) and 52.8% (oronasal) of the time. They used two respiratory phase patterns across a set of four trials 35.3% (nasal) and 42.5% (oronasal) of the time, and three patterns 5.3% (nasal) and 4.7% (oronasal) of the time (Figure 2). Regarding post‐swallow respiratory direction in isolation, participants consistently exhaled after swallowing 83.9% (nasal) and 72.4% (oronasal) of the time across a set of four trials of the same swallowing condition. In contrast, they consistently inhaled after swallowing 1.6% (nasal) and 3.9% (oronasal) of the time across a set of four trials from the same swallowing condition. Participants were inconsistent across sets (i.e., displayed both post‐swallow inhalation and exhalation) 14.5% (nasal) and 23.6% (oronasal) of the time. The full dataset pertaining to test–retest reliability is supplied as Supporting Information.

## 4 DISCUSSION

### 4.1 Intra‐ and inter‐rater reliability for respiratory phase patterns

When evaluating respiratory phase patterns across the two masks, it is essential to first consider the reliability of the data within and between raters. The data suggest higher inter‐rater reliability according to oronasal mask trials compared to nasal mask trials. It is possible (but not certain) that this resulted from genuine differences in how the two masks measured respiratory airflow, impacting data clarity. Given our findings of generally high *intra*‐rater reliability on both oronasal and nasal trials, the difference in *inter*‐rater reliability suggests there was more ambiguity in the nasal waveform, which caused disagreement between the raters. The greatest source of disagreement between raters was the distinction between the EX–EX and IN–EX patterns in the nasal dataset; raters disagreed on 87 trials across all swallowing tasks, compared to only 16 disagreements of the same kind using oronasal data. Therefore, the low intra‐rater reliability in the nasal‐only dataset was most likely caused by a persisting lack of clarity regarding the difference between SNRF and respiration. This was reflected by the inconsistent SNRF measurements between raters in the nasal data. This inconsistency occurred despite the measures taken to aid interpretation in this study. Determining a respiratory phase pattern is theoretically unambiguous, as ratings are based on a binary distinction between inhalation and exhalation, which should clearly differ on a spirometry waveform; SNRF is the most likely additional factor to hinder clear interpretation. Of note, continuous swallows from the oronasal mask had lower intra‐rater reliability than any other condition. There are several possible explanations for this, including decreased clarity of the respiratory waveform in cases where multiple swallows occurred within the same ingestive cycle; increased artifact due to pressure changes from the straw used within the closed facemask system; or less obvious respiratory patterning due to participants taking quicker and shallower breaths between swallows, which are less likely to be clearly observed via the respiratory waveform or abdominal kinematic measures.

### 4.2 Frequency of respiratory phase patterns across masks

The predominance of EX–EX patterns in this study supports existing literature (Hopkins‐Rossabi et al., 2019; Martin‐Harris et al., 2005). However, increased estimates of EX–EX patterning using nasal flow compared to oronasal suggest previous results could potentially have been influenced by use of nasal data in isolation. While oral airflow had a relatively minor influence on non‐ingestive swallowing, as evidenced by similar estimates of respiratory phase patterns across the nasal and oronasal masks, findings show benefit in accounting for combined oral and nasal respiratory flow when assessing ingestive swallowing.

Differences between nasal and oronasal estimates were particularly evident in the liquid swallowing conditions. In both the 10 mL and continuous drinking conditions, EX–EX patterning occurred less frequently according to oronasal data compared to nasal. On 10 mL water trials, the decrease in EX–EX swallows was countered by increased IN–EX swallows, while during continuous drinking there were increased rates of both EX–IN and IN–IN. Robust physiological measures of lung volume are still needed to establish whether these findings reflect greater sensitivity in the oronasal measures (and thus, greater accuracy to respiratory flow), or whether they simply caused ambiguity in the data resulting from SNRF‐related artefact. It is possible that adding oral flow to nasal flow increased detection of pre‐swallow inhalation (i.e., IN–EX) on the 10 mL water trials; although it could have resulted instead from SNRF‐related ambiguity, this seems unlikely since the same pattern would likely have been observed in the continuous drinking condition.

During continuous drinking, addition of oral to nasal flow changed estimated *post*‐swallow flow direction, evidenced by the lower rates of EX–EX and higher rates of EX–IN and IN–IN. More frequent post‐swallow inhalation was expected, as this is a known alteration during continuous drinking (Dozier et al., 2006; Hirst et al., 2002; Martin et al., 1994; Wheeler Hegland et al., 2011). We theorize that the difference in estimates during continuous drinking resulted from the increased sensitivity of the oronasal facemask, allowing detection of subtle differences in respiratory airflow that was not possible using the nasal mask. Again, it remains unknown whether this increase in sensitivity translates to increased *accuracy*, or if it simply impeded interpretation of the data. Further physiological investigation is required to confirm the validity of these findings. It is also possible that the use of a straw influenced oronasal measurements more than nasal, as the oronasal facemask is more likely to detect straw‐related pressure changes due to the oral cavity and straw being sealed within the mask; it is unlikely to cause such an effect with the nasal mask, as it is not contained within the closed mask system. However, the finding of only minor differences between cup and straw trials (using the nasal mask only) suggested that use of the straw is unlikely to have had any considerable influence on respiratory phase patterns in this study.

According to the nasal mask data, we observed a considerably higher frequency of EX–EX patterning during continuous drinking compared to previous studies (Dozier et al., 2006; Lederle et al., 2012; Wheeler Hegland et al., 2011), signalling that EX–EX may be overestimated in our continuous drinking data. When comparing the current estimates to those of previous studies of continuous drinking, the closest similarity was between our oronasal estimates and those presented by Wheeler Hegland et al. (2011). Despite limited physiological evidence in the current study, there are several notable similarities in methods across the two studies which may suggest greater accuracy in our oronasal estimates compared to the nasal estimates. For example, this and the Wheeler Hegland et al. (2011) study both assessed fluid trials of a similar volume, delivered through a straw. Additionally, both utilised respiratory belt measures, thereby reflecting total respiration (i.e., oral and nasal), although this comparison is somewhat limited due to the lack of abdominal kinematic data and lung volume measures in the current study.

Although our results support the influence of oral airflow on respiratory phase pattern estimates, a considerable limitation of the study is that nasal and oronasal data were collected using different masks at different time points (i.e., different swallowing trials). As such, the respiratory phase patterns measured from nasal and oronasal data could not be directly compared within a given trial as in previous work (Cross et al., 2024). Differences in the frequency of each respiratory phase pattern across the oronasal and nasal masks may partly reflect behavioural differences across asynchronous trials, rather than a difference in how the same pattern was measured. Importantly, the similar frequencies of each respiratory phase pattern observed across the two masks in the non‐ingestive conditions suggest that neither mask directly caused respiration or swallowing to be altered. However, the more drastic differences seen in the ingestive conditions could reflect a greater influence of the mask when eating and drinking. It is impossible to rule out measurement error or changes in participant behaviour as the result of wearing either mask. The cause of discrepancies between nasal and oronasal estimates remains unknown, and the validity of each tool cannot be determined based on current data. There is also a possibility that our kinematic measures were less sensitive due to the exclusion of chest kinematic data from analysis. Given that the abdominal band should register a degree of rib cage displacement (Banzett et al., 1995) and respiratory phase patterns are primarily assessed based on tidal airflow, we think it unlikely that results were influenced greatly; however, distinction between respiratory flow and SNRF may have been less sensitive, and we cannot guarantee that results were not impacted to some degree.

### 4.3 SNRF

We consider ambiguity regarding SNRF to be the most likely cause of disagreement within and between raters. Intra‐ and inter‐rater reliability for SNRF data indicated that the raters were relatively consistent at judging whether a SNRF event did or did not occur on any given swallowing trial. However, the reduced reliability seen when the data are further stratified demonstrates the challenges associated with distinguishing between SNRF and respiratory flow given the lack of clear definition. Regarding the position of SNRF, data were analysed according to whether an event was recorded at the beginning or end of the respiratory–swallowing pause, or both. There was frequent disagreement between ratings due to one rating identifying SNRF both at onset‐ and offset‐ of the respiratory–swallowing pause, with the other only identifying SNRF in one position. For ratings of SNRF direction, reliability was calculated based on each individual SNRF event, and results were influenced by several factors. For one, on some trials, the presence of SNRF was disputed, i.e., one rating identified a SNRF event as present and the other identified no SNRF event; therefore, there was an inherent lack of agreement between the two ratings. An example of this is the observation that the two raters had difficulty distinguishing between EX–EX and IN–EX patterns on numerous trials in the nasal data; this suggests that ambiguity was caused by the presence of a brief inward flow event at the very beginning of the respiratory–swallowing pause. This demonstrates the need to determine the physiology underlying this ambiguous airflow and to distinguish respiratory from non‐respiratory flow surrounding the respiratory–swallowing pause. Although less common, there were cases in which both ratings identified SNRF, but the two ratings disagreed about the direction of airflow. This was likely due to a noisy, unclear waveform caused by the increased sensitivity of flow measures, resulting from the fully sealed masks used in this study, which complicated analysis. As previously discussed, the distinction between respiratory flow and SNRF, as well as identification of the characteristics of SNRF, may have been enhanced if chest kinematic data had been available due to its greater sensitivity compared to abdominal data on its own.

Despite the issues with reliability of SNRF measures, the findings largely support previous evidence that SNRF can occur at the beginning of the respiratory–swallowing pause and that it can consist of inward or outward flow (Cross et al., 2024). This study suggests a higher frequency, with observation of SNRF in up to 97% of swallows, compared to previous reports of 67–74% (Brodsky et al., 2012). The increased prevalence of SNRF is likely due to the identification of outward flow events, events occurring at the beginning of the respiratory–swallowing pause, and the increased sensitivity of our closed system capable of detecting all flow. We theorize that use of the fully enclosed masks in this study detected low amplitude airflow (respiratory and non‐respiratory), allowing more SNRF events to be identified than with the nasal cannula method used previously (Brodsky et al., 2012; Martin‐Harris et al., 2003). However, these findings cannot be confirmed without additional physiological evidence, and thus, should be interpreted somewhat cautiously.

Although our data support the notion that SNRF is more broadly presenting than previously thought, SNRF at the end of the respiratory–swallowing pause appears to be most common, supporting previous studies (Brodsky et al., 2012; Martin‐Harris et al., 2003). Videofluoroscopic investigations suggest that this is caused by re‐opening of the pharynx after swallowing (Brodsky et al., 2012; Paydarfar et al., 1995). The lack of videofluoroscopic data in the current study precludes the integration of SNRF findings with biomechanical evidence. However, we theorize that outward airflow at the beginning of the respiratory–swallowing pause is caused by ejection of air from the pharynx during contraction in preparation for swallowing. This may be caused by movement of the velum toward the posterior pharyngeal wall, thereby ejecting air from the nasal cavity. Another contributing factor may be elevation and constriction of the larynx and pharynx during periods of thoracic fixation, causing air within the dead space of the pharynx to be expelled with movement of these structures (i.e., since the thorax is fixed, excess air can only exit the nose). We expect that outward airflow at the end of the respiratory–swallowing pause is caused by a contraction of the lungs during swallowing which leads to a non‐respiratory expulsion of air when the larynx un‐valves. This theory is based on the observation of continuous abdominal movement via the respiratory belt, suggesting abdominal tightening during swallowing creates pulmonary pressure that releases immediately after the larynx is un‐valved. We theorize that inward flow at onset‐pause occurs during swallows initiated at lower‐than‐typical lung volumes. It has been posed that swallowing is initiated at mid–low lung volumes, which allows easy laryngeal elevation for airway closure (McFarland et al., 2016). However, if lung volumes are lower than optimal, e.g., after maximal exhalation, the larynx may be fixed in a low position. A small influx of air may be needed to re‐inflate the lungs to a mid–low volume, facilitating laryngeal elevation.

Our findings of increased frequency of SNRF, outward as well as inward airflow, and SNRF at the onset of the respiratory–swallowing pause provide new insights into RSC. However, further research is required, with a particular need for simultaneous respiratory and videofluoroscopic data to identify the swallowing physiology causing SNRF. This development also presents a new challenge for ensuring accurate interpretation of respiratory phase patterns; determining the physiology underlying SNRF will be essential for distinguishing between respiratory and non‐respiratory flow surrounding swallowing in future studies. Additionally, defining the duration and amplitude of SNRF (and thus, distinguishing it from respiratory flow as well as artifact in the flow signal) is a challenging but necessary task for improving understanding of SNRF. This is particularly important given the suboptimal reliability of both respiratory phase patterns and SNRF in the current study, despite the addition of tools to clarify measurements.

### 4.4 Test–retest reliability

Our results provide further evidence that individuals do not necessarily use the same respiratory phase pattern consistently across multiple trials of the same swallowing condition, extending previous incidental findings (Cross et al., 2024). The notion of variability is supported by previous reports of individuals using multiple patterns within the same or similar swallowing conditions (Hardemark Cedborg et al., 2009; Wheeler Hegland et al., 2011, 2009); however, findings cannot be more closely compared, due to a lack of specific test–retest reliability measures in previous studies. In the current study, a degree of variability in patterning was evident over repeated trials according to both nasal and oronasal data. Consistent use of one respiratory phase pattern across all four trials of the same bolus condition was seen just over half of the time, while at least two patterns were used across a set of four trials >40% of the time. One previous study reported that 80% of participants used both EX–EX and EX–IN patterns during discrete bolus swallows, with more crossover of IN–EX and IN–IN patterns also implied (Wheeler Hegland et al., 2009). However, the authors did not provide information regarding exactly how many patterns were used by any given individual. Additionally, despite all trials being completed on discrete boluses, they did not provide specific information on the patterns used for differing bolus types (e.g., liquid and paste), meaning variability seen in their sample may have been impacted by the use of varying bolus types. No individual in the current study used all four respiratory phase patterns within the same bolus type. Use of four patterns has been reported in continuous water drinking (Wheeler Hegland et al., 2011), though again, the authors collapsed the two conditions (cup and straw), which may have influenced this finding.

According to both nasal and oronasal data, there were two common scenarios: participants were likely to use either (a) EX–EX consistently across a set of four trials, or (b) a combination of any four respiratory phase patterns across a set of four trials. It was uncommon for a participant to consistently use just one pattern *other than* EX–EX across a set of four trials of the same condition. The rare occurrence of consistent EX–IN or IN–IN patterning over all four trials was only ever observed during continuous drinking. Additionally, when a combination of patterns was displayed across four swallows from the continuous drinking trial, there was always at least one swallow that was followed with inhalation, consistent with findings of pattern variability (Wheeler Hegland et al., 2011) and increased post‐swallow inhalation (Dozier et al., 2006; Wheeler Hegland et al., 2011) during continuous drinking.

There was considerably less variability in post‐swallow respiratory flow direction on its own than in full respiratory phase patterns (i.e., including pre‐ and post‐swallow). Post‐swallow exhalation, which occurred consistently in most sets of four trials, is thought to contribute to airway safety, minimising aspiration risk (Preiksaitis & Mills, 1996) by clearing residue from the airway entrance (Preiksaitis et al., 1992). The finding that the direction of post‐swallow airflow is relatively stable suggests inconsistency in full respiratory phase patterns is largely caused by changing *pre‐swallow* airflow direction. Therefore, pre‐swallow airflow appears to be the factor that contributes most to within‐individual behavioural variability. Pre‐swallow lung volume is thought to optimise biomechanics for swallowing (McFarland et al., 2016). Thus, flexibility in pre‐swallow respiratory flow direction may serve to establish optimal lung volumes, facilitating efficient swallowing. Consequently, whilst exhaling post‐swallow is likely to support airway clearance, it is also possible that passive exhalation is simply the most likely physiological response after swallowing, as any further inflation of the lungs is deemed unnecessary. Although rare, the majority of swallows followed by inhalation were either continuous (which is unsurprising given known behavioural differences; Dozier et al., 2006; Wheeler Hegland et al., 2011) or non‐ingestive (spontaneous or cued dry).

The apparent inconsistency in pre‐swallow airflow direction suggests a degree of flexibility in respiratory patterning leading up to swallowing. Depending on an individual's lung volume in the build‐up to swallowing, they may exhale or inhale as required to facilitate optimal lung volumes, setting up optimal swallowing physiology and thus, efficient and safe swallowing. Pre‐swallow respiratory direction may further contribute to safety by ensuring optimal lung volumes for bolus expulsion if needed (Wheeler Hegland et al., 2009). The flaw in this theory is that respiratory phase patterns have previously been shown to have little influence on lung volumes (Wheeler Hegland et al., 2009), though further investigation is warranted to define the relationship between respiratory flow, especially pre‐swallow, and other respiratory characteristics such as lung volume. It seems relatively likely based on our data that post‐swallow exhalation remains stable for airway safety, though the physiological role of pre‐swallow airflow direction is yet to be fully understood.

The fact that variability was observed in both the nasal and oronasal datasets indicates that it is characteristic of RSC, rather than a result of measurement technique. As such, variability (i.e., test–retest reliability) should be considered in future investigations of respiratory phase patterning, in both healthy and disordered populations. However, some noteworthy differences were observed between the nasal and oronasal data. While consistent use of one respiratory phase pattern was slightly more common (difference of 7%) when measured by the nasal mask, consistent post‐swallow exhalation was observed 12% less frequently when measured by the oronasal mask. Although these findings could be the result of measurement differences across trials or measurement error, they could also suggest behavioural inconsistency in the presence of mouth breathing. Importantly, the reduced frequency of post‐swallow exhalation in the oronasal data may point toward greater risk during swallowing in mouth breathers, particularly when dysphagic.

### 4.5 Implications of findings

This work has provided essential new insights into healthy RSC, which have the potential to aid comparisons between healthy and dysphagic individuals, as well as influence approaches to RSC assessment and treatment. Comparison of nasal and oronasal airflow measures has demonstrated that estimates of respiratory phase patterns are altered when oral and nasal respiratory airflow are assessed simultaneously. These findings show that all respiration (regardless of its route through the upper airway) should be accounted for when assessing respiratory phase patterns. Isolated nasal measures are unlikely to fully reflect respiratory behaviour in the context of swallowing; this is likely to be more pertinent in individuals prone to mouth breathing, and as such, accounting for oral airflow may be of greater importance when assessing this population. Direct assessments of oral and nasal flow, as used in this study, are unlikely to be practical or warranted in all situations; however, there is benefit in incorporating tools that represent total respiration, such as kinematic rib cage and abdominal measures. Given the influence of respiratory flow on lung volumes, and the likely influence of lung volume on swallowing physiology (McFarland et al., 2016), it is essential that respiration be fully and accurately measured in investigations of RSC. Of course, the accuracy of the current instrumentation remains unknown, as does the extent to which its increased sensitivity impeded or supported measurement accuracy.

These findings also expand our understanding of SNRF, providing further evidence that it can occur at the beginning of the respiratory–swallowing pause as well as the end, and can consist of outward flow as well as inward. Although we have theorized the underlying physiology, investigations using refined measures are needed to define the biomechanical events causing various presentations of SNRF. Increased identification of SNRF likely occurred due to use of enclosed, sealed masks, resulting in greater sensitivity to airflow; despite the benefit arising from this, our dataset was also negatively impacted by the ambiguity that resulted. It is essential that SNRF is better defined, to allow accurate distinction between respiratory and non‐respiratory flow, thereby reducing ambiguity and disagreement, and facilitating greater accuracy in future studies. Even studies that do not set out to assess SNRF need to consider its potential influence on respiratory flow estimates, particularly when using highly sensitive instrumentation.

Through targeted assessment of test–retest reliability of respiratory phase patterns, this study has demonstrated variability in healthy individuals’ respiratory behaviour across swallows. Therefore, healthy swallowing does not appear reliant on use of a fixed pattern. The relationship between variability in healthy individuals and outcomes in eventual disease is yet to be determined. Given the apparent detrimental effect of dyscoordinated respiratory phase patterning on swallowing safety in patient populations (Brodsky et al., 2010; Hopkins‐Rossabi et al., 2021; Troche et al., 2011), it could be that inconsistent patterning is of minimal consequence to healthy individuals, but becomes problematic after onset of dysphagia. It is also entirely possible that premorbid variation from ‘optimal’ patterning predisposes certain individuals to worse swallowing‐related outcomes, regarding both safety and efficiency, though this requires targeted investigation. Emerging treatments targeting RSC, including respiratory phase patterning, have considerable potential to benefit individuals with dysphagia secondary to various conditions (Curtis, Dakin et al., 2020, 2020; Martin‐Harris et al., 2015). Based on our findings, it may be advantageous to consider the variability in healthy individuals’ behaviour when designing and implementing therapeutic approaches.

### 4.6 Conclusion

This study supports previous findings that adding oral to nasal respiratory airflow data alters estimates of respiratory phase patterns surrounding swallowing, particularly when drinking fluids. Although it remains unknown whether this reflects greater accuracy, results suggest that it is necessary to account for all respiratory airflow (i.e., oral and nasal) when assessing respiratory phase patterns. This study has also provided further evidence that SNRF can occur more broadly than previously recognized in the literature, consisting of inward or outward flow at the beginning or end of the respiratory–swallowing pause. Further work is required to define the physiology underlying non‐respiratory flow, and to better distinguish it from respiratory flow or artefact to improve the accuracy of ongoing work. Finally, the findings have provided additional evidence that healthy individuals use respiratory phase patterns less consistently than previously implied. Inconsistency appears to be caused most often by variability in the direction of airflow immediately prior to swallowing, given that post‐swallow direction, particularly exhalation, appears to be relatively stable. We theorize that flexibility in pre‐swallow respiratory flow direction serves to adapt lung volumes as necessary to support swallowing biomechanics, while relatively consistent post‐swallow exhalation protects against aspiration. Ongoing research is needed to determine the exact relationship between respiratory phase patterns and lung volumes.

## AUTHOR CONTRIBUTIONS

Elizabeth Cross, Esther Guiu Hernandez and Phoebe Macrae contributed to conception and design of this study. Elizabeth Cross, Esther Guiu Hernandez and Phoebe Macrae contributed to acquisition, analysis and interpretation of data. Elizabeth Cross drafted the manuscript, and all authors revised it critically for important intellectual content. All authors approved the final version of the manuscript; agree to be accountable for all aspects of the work in ensuring that questions related to the accuracy or integrity of any part of the work are appropriately investigated and resolved; all persons designated as authors qualify for authorship; and all those who qualify for authorship are listed.

## CONFLICT OF INTEREST

None declared.

## Supporting information




Data: respiratory phase patterns, SNRF, test‐retest reliability

## Data Availability

The data that support the findings of this study are available as Supporting Information.
